# Association between Proinflammatory Cytokines and Lung Cancer Risk: A Case-Cohort Study from a Community-Based Prospective Cohort

**DOI:** 10.3390/cancers15235695

**Published:** 2023-12-03

**Authors:** Eun Young Park, Eunjung Park, Taiyue Jin, Min Kyung Lim, Jin-Kyung Oh

**Affiliations:** 1 Department of Preventive Medicine, Korea University College of Medicine, Seoul 02841, Republic of Korea; eunyoungpark2022@gmail.com; 2 Department of Cancer Control and Population Health, National Cancer Center Graduate School of Cancer Science and Policy, Goyang 10408, Republic of Korea; eunjungpark@ncc.re.kr; 3 Division of Cancer Prevention, National Cancer Control Institute, National Cancer Center, Goyang 10408, Republic of Korea; taewol@ncc.re.kr; 4 Department of Social and Preventive Medicine, Inha University College of Medicine, Incheon 22212, Republic of Korea; mickey@inha.ac.kr

**Keywords:** lung cancer, proinflammatory cytokines, IL-6, IL-1β, IFN-γ, case-cohort study, cohort

## Abstract

**Simple Summary:**

Recent studies have shed light on alterations in the proinflammatory tumor microenvironment as a significant carcinogenic mechanism. We carried out a case-cohort study to investigate associations between proinflammatory cytokines and lung cancer risk, considering histological types in the Korean general population (lung cancer cases: 136, subcohort: 822). Serum levels of proinflammatory cytokines (i.e., IL-6, TNF-α, IL-1β, IFN-γ, and IL-10) were measured using Quantikine^®^ ELISA. A Cox proportional-hazards regression analysis was conducted. Serum levels of IL-6, IL-1β, and IFN-γ were associated with lung cancer risk. IL-6 was associated with lung cancer risk, regardless of the histological type. IL-1β and IFN-γ had associations with adenocarcinoma and squamous-cell carcinoma, respectively. This study, targeting an Asian population, adds causality to association between proinflammatory cytokines and lung cancer risk by replicating previous findings. Furthermore, it suggests that specific cytokines, depending on the histological type of lung cancer, may serve as potential biomarkers.

**Abstract:**

Recent studies have shed light on alterations to the proinflammatory tumor microenvironment as a significant carcinogenic mechanism. Despite previous studies on associations between proinflammatory cytokines and lung cancer risk, few studies have been conducted in Asian populations. This study aimed to investigate associations between proinflammatory cytokines and lung cancer risk, considering histological types, in the Korean general population. We carried out a case-cohort study on the Korean National Cancer Center Community (KNCCC) cohort (lung cancer cases: 136, subcohort: 822). Pre-diagnostic serum levels of proinflammatory cytokines (i.e., IL-6, TNF-α, IL-1β, IFN-γ, and IL-10) were measured using Quantikine^®^ ELISA. A Cox proportional-hazards regression analysis was conducted. In this study, serum levels of IL-6, IL-1β, and IFN-γ were associated with lung cancer risk. IL-6 was associated with lung cancer, regardless of the histological type. IL-1β had an association only with adenocarcinoma, while IFN-γ had an association only with squamous-cell carcinoma. This study shows associations between serum levels of IL-6, IL-1β, and IFN-γ and lung cancer risk, underscoring the potential of these cytokines to act as risk biomarkers. The utilization of these biomarkers for risk prediction may hold the promise of facilitating the identification of the high-risk population.

## 1. Introduction

Worldwide, lung cancer is the most common cause of cancer-related deaths, constituting 21.5% of all deaths in men and 13.7% of them in women, and it accounts for 14.3% of new cancer cases among men and 8.4% among women [1]. In the Republic of Korea, during 2020, the incidence of lung cancer reached 19,657 cases (15.0%) in men, making it the predominant malignancy among men, and 9292 cases (7.9%) in women, ranking it as the third-most-common cancer among women. Among the 28,949 cases of lung cancer in 2020, classified under the 10th revision of the International Classification of Diseases (ICD-10) with the code C34, carcinomas were the prevailing histological entity, constituting a substantial 89.9%, while sarcomas made up 0.1%. Of the carcinomas, adenocarcinoma was the most prominent subtype, representing 50.6% of the cases, followed by squamous-cell carcinoma at 19.7% and small cell carcinoma at 10.5%. Notably, adenocarcinoma demonstrates a sustained upward trend, particularly among women, whereas the new cases of squamous-cell carcinoma and small cell carcinoma are showing declines [2].

A widely well-established risk factor for lung cancer is cigarette smoking, while environmental factors like air pollution, asbestos, metals such as cadmium, beryllium, and chrome, etc., ionizing radiation, and polycyclic aromatic hydrocarbons (PAHs) have also been identified as contributors to its pathogenesis [3]. Recent studies have shed light on the role of inflammation as a significant carcinogenic mechanism, with alterations in the proinflammatory tumor microenvironment (TME), including heightened levels of interleukin 1β (IL-1β), interleukin 6 (IL-6), interleukin 8 (IL-8), interleukin 10 (IL-10), tumor necrosis factor-α (TNF-α), interferon gamma (INF-γ), reactive oxygen species (ROS), nitric oxide (NO), and reactive nitrogen species (RNS), known to promote cancer initiation, progression, and metastasis [4,5]. Previous studies have suggested associations between serum proinflammatory cytokines and lung cancer risk. A study conducted in Australia and Sweden found that elevated IL-6 levels were linked to higher lung cancer risk in former and current smokers. Similarly, increased IL-8 levels were associated with a greater risk in former and current smokers, but not in never-smokers [6]. Another study by Pine et al. reported similar IL-6 and IL-8 associations with the lung cancer risk across racial groups, particularly among African Americans and European Americans. Notably, elevated serum levels of IL-1β, IL-10, and TNF-α were linked to lung cancer risk only in the African American population [7]. However, most of previous studies were performed in Western populations and did not consider the histological types of lung cancer. Thus, replication of previous findings is needed in other populations, especially Asian populations.

We carried out a case-cohort study on the Korean National Cancer Center Community (KNCCC) cohort to evaluate the associations between proinflammatory cytokines and lung cancer risk, considering histological types, using serum samples collected before cancer diagnosis.

## 2. Materials and Methods

### 2.1. Case-Cohort Study Design and Study Population

The KNCCC cohort, conducted between 1993 and 2010, was a community-based prospective study that was performed in order to explore the risk factors for cancers and the protective factors associated with their prevention [8]. The 16,304 participants aged over 30 entered the cohort in Changwon, Chuncheon, Chungju, Sancheong, and Haman. Detailed baseline information regarding demographics and lifestyle and environmental factors were obtained through a structured questionnaire. Additionally, physical examination and laboratory assessments were carried out, and blood and urine samples were obtained at cohort enrollment.

The obtained serum samples were stored at −70 °C (between 1993 and 2008) and were preserved at −140 °C (between 2009 and 2010). The collected data were linked to national cancer incidence data from the Korea Central Cancer Registry, with follow-up until 31 December 2017.

This study received approval from the KNCC institutional review board (no. NCC2020-0203), and all participants provided written informed consent. This case-cohort study was reported according to the Strengthening the Reporting of Observational Studies in Epidemiology (STROBE) guidelines.

To investigate the associations between serum levels of proinflammatory cytokines and lung cancer risk, a case-cohort study was designed. Of 16,304 men and women, we excluded participants without a serum sample (n = 2059), participants with missing data for other covariates (e.g., education achievement, cigarette smoking status, alcohol consumption status, body mass index [BMI]) (n = 4628), and participants with a follow-up period of less than 1 year to exclude undiagnosed cancer (n = 446).

**Figure 1:**
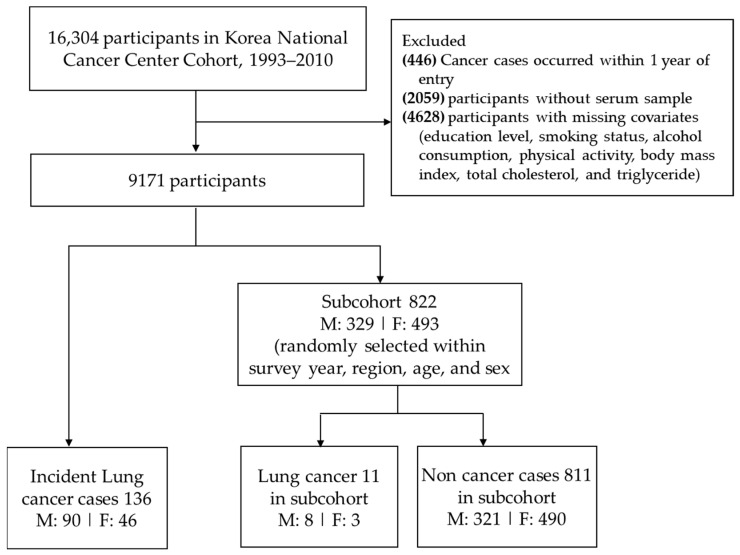
Study participant selection process.

A representative subcohort of 822 participants was randomly selected by survey year, region, age, and sex from the eligible population who participated in the cohort between 2001 and 2010 (n = 9171). As a result, 136 lung cancer cases (124 cases plus 11 cases in the subcohort) and 822 subcohort participants (11 cases in the subcohort) were included in this study (Figure 1).

### 2.2. Outcome Definition

We defined the outcome as primary lung cancer coded C33 or C34 by the ICD-10.

### 2.3. Measurements of Serum Proinflammatory Cytokines

Serum levels of proinflammatory cytokines were measured using Quantikine^®^ ELISA. The intra- and inter-assay precision are reported as follows: coefficient of variation (CV, %): IL-6 (1.7–4.4/2.0–3.7), TNF-α (4.2–5.2/6.8–8.7), IL-1β (2.8–8.5/4.1–8.4), IFN-γ (2.6–4.7/2.7–7.8), IL-10(1.7–5.0/5.6–7.6). The average recoveries are 93% (IL-6), 107% (TNF-α), 95%(IL-1β), 102% (IFN-γ), and 100%(IL-10), respectively. The limits of detection (LODs) are 0.01 pg/mL (IL-6), 0.01 pg/mL (TNF-α), 0.01 pg/mL (IL-1β), 0.01 pg/mL (IFN-γ), and 0.01 pg/mL (IL-10), respectively.

### 2.4. Statistical Analyses

We compared differences in demographic characteristics between lung cancer cases and subcohorts at baseline: age, sex, region, year of enrollment, education achievement (elementary school or less, middle school, high school, or college or higher), cigarette smoking status (never smokers, former smokers, current smokers), pack-years (continuous variable), alcohol consumption status (non-drinkers, <24 g per day, ≥24 g per day), and body mass index (BMI, continuous variable).

Serum levels of proinflammatory cytokines were natural log-transformed due to their right-skewed distribution and were categorized into quartiles aligning with the proinflammatory cytokine distribution within the subcohort (alternatively, when levels exceeded or were equal to 50% of the LOD, they were dichotomized into two groups using the median value.), facilitating the derivation of hazard ratios (HRs) that are easily interpretable and allowing for the identification of potential non-linear associations.

To examine the associations between serum levels of proinflammatory cytokines and lung cancer risk, a Cox proportional-hazards regression analyses was conducted, the attained age was applied as a time scale [9], and the weighted method by Barlow was used for our case-cohort design [10]. There was no violation of the proportional-hazards assumption. The validity of the assumption was assessed by calculating Schoenfeld residuals. 

Three Cox proportional-hazards models were applied: the first model was unadjusted; the second model was adjusted for sex; and the third model included the further year of enrollment, region, education achievement, cigarette smoking status, number of pack-years, alcohol consumption status, and BMI. The *p* for trend was produced by linear regression through regressing the hazard ratios (HRs). Furthermore, a stratified analysis was conducted to assess whether the associations differ, according to the cigarette smoking status (never-smokers and ever-smokers).

We performed a subgroup analysis to assess the lung cancer risk by major histologic types (i.e., M-code: squamous-cell carcinoma (8050–8078, 8083–8084), adenocarcinoma (8140, 8211, 8230–8231, 8250–8260, 8323, 8480–8490, 8550–8551, 8570–8574, 8576) and small-cell carcinoma (8041–8045, 8246)). 

All statistical analyses were performed as two-tailed tests with a significance level of *p* < 0.05) using SAS version 9.4 (SAS Institute, Cary, NC, USA).

## 3. Results

### 3.1. Baseline Characteristics of the Study Participants

Table 1 presents the differences in the demographic characteristics of the 136 lung cancer cases and the 822 subcohort cases. The median follow-up periods were 6.79 years for lung cancer cases (interquartile range [IQR], 4.00–9.45) and 11.43 years for the subcohort (IQR, 8.48–13.88), and the person-times were 939.88 years for lung cancer cases and 9004.12 years for the subcohort. Lung cancer cases had higher proportions of men, the elderly, low-educated persons, cigarette smokers, and drinkers than the subcohort, whereas the subcohort had higher proportions of obesity and an education level of high school or greater than lung cancer cases.

The serum levels of proinflammatory cytokines are presented in Table 2. The median IL-6, TNFα, IL-1β, and IFN-γ values were higher in lung cancer cases than in the subcohort.

### 3.2. Associations between Serum Levels of Proinflammatory Cytokines and the Lung Cancer Risk

**Figure 2:**
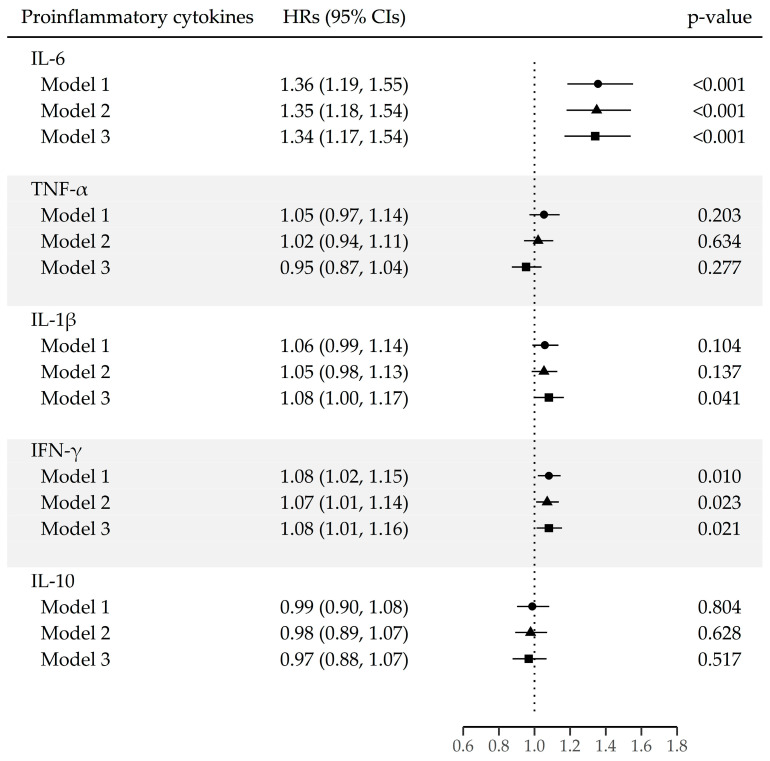
Lung cancer risk for each unit increase in the natural log-transformed proinflammatory cytokines.

Figure 2 shows the associations between serum levels of proinflammatory cytokines and the lung cancer risk. The risk of lung cancer increased for each unit increase in the natural log-transformed IL-6 (HR [95% CI]: 1.34 [1.17–1.54]), and IFN-γ (HR [95% CI]: 1.08 [1.01–1.16]).

When applying the Cox proportional-hazards model with categorized variables, the risk increased from the second quartile of serum IL-6 (2nd quartile (HR [95% CI]: 8.54 [3.30–22.11]), 3rd quartile (HR [95% CI]: 5.56 [2.17–14.27], 4th quartile (HR [95% CI]: 9.51 [3.72–24.30]). In addition, the lung cancer risk was associated with higher levels of IL-1β and IFN-γ than the LOD value (HR [95% CI]: 1.61 [1.11–2.32], 1.85 [1.26–2.72], respectively) (Table 3).

After stratification analysis with the cigarette smoking status, the effect of serum proinflammatory cytokines on the lung cancer risk was more prominent in never-smokers. IL-6, IL-1β, and IFN-γ had consistent associations with the lung cancer risk (HR [95% CI]: 1.31 [1.04–1.66], 1.25 [1.06–1.48], 1.23 [1.07–1.42], respectively), whereas, in ever-smokers, only IL-6 had an association with the lung cancer risk (HR [95% CI]:1.38 [1.16–1.64]) (Table 4).

The hazard ratios (HRs) are presented for each unit increase in natural log-transformed proinflammatory cytokines, derived from Cox proportional-hazards models. Model 1 was unadjusted; Model 2 was adjusted for sex; Model 3 was further adjusted for year of enrollment, region, education achievement, cigarette smoking status (never, former, and current), number of pack-years (continuous variable), alcohol consumption status (non-drinkers, <24 g per day, and ≥24 g per day), and BMI (continuous variable). 

The following abbreviations are used: IL-6, interleukin 6; TNF-α, tumor necrosis factor-α; IL-1β, interleukin; IFN-γ, interferon gamma; and IL-10, interleukin 10.

### 3.3. Associations between Serum Levels of Proinflammatory Cytokines and Lung Cancer Risk by Major Histologic Type

**Figure 3:**
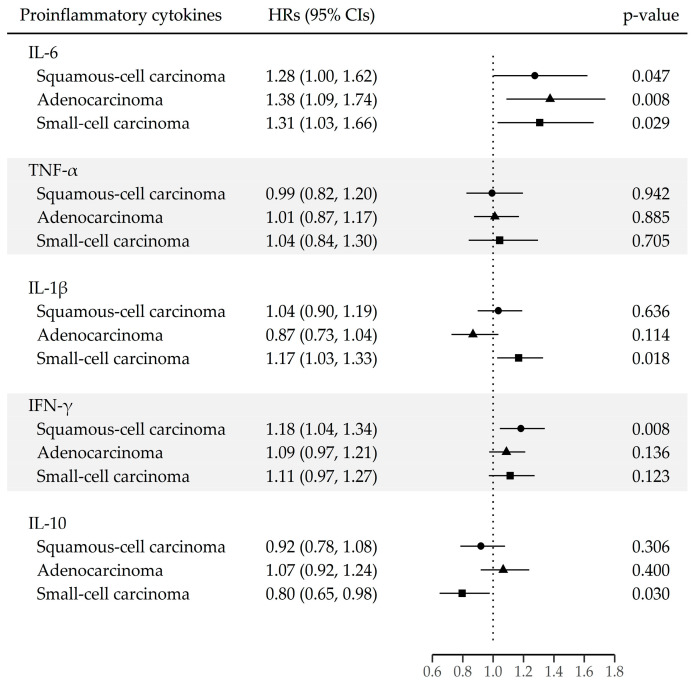
Lung cancer risk for each unit increase in natural log-transformed proinflammatory cytokines by major histologic type. The hazard ratios (HRs) are presented for each unit increase in natural log-transformed proinflammatory cytokines, derived from Cox proportional-hazards models. Values are adjusted for sex, year of enrollment, region, education achievement, cigarette smoking status (never, former, and current), number of pack-years (continuous variable), alcohol consumption status (non-drinkers, <24 g per day, and ≥24 g per day), and BMI (continuous variable). Abbreviations: IL-6, interleukin 6; TNF-α, tumor necrosis factor-α; IL-1β, interleukin; IFN-γ, interferon gamma; IL-10, interleukin 10.

Figure 3 shows that IL-6 was associated with lung cancer, regardless of the histological type (HR [95% CI]: squamous-cell carcinoma, 1.28 [1.00–1.62]; adenocarcinoma, 1.38 [1.09–1.74]; small-cell carcinoma, 1.31 [1.03–1.66], respectively). IL-1β had an association only with small-cell carcinoma (HR [95% CI]: 1.17 [1.03–1.33], while IFN-γ had an association only with squamous-cell carcinoma (HR [95% CI]: 1.18 [1.04–1.34]).

## 4. Discussion

Our study demonstrates the association between serum levels of IL-6, IL-1β, and IFN-γ and the lung cancer risk. These associations persist even after adjustment for various potential confounding variables and are also strengthened by stratification for cigarette smoking status. Furthermore, in this study, the utilization of serum samples collected prior to the diagnosis of lung cancer negates the possibility of reverse causation, which implies that the presence of undiagnosed lung cancer might have influenced the serum levels of proinflammatory cytokines. It is important to note that we excluded cancer occurrences within 1 year of follow-up, and since the serum samples for measurement were collected a median of 6.79 years before the diagnosis of lung cancer, the robustness of the association between serum levels of proinflammatory cytokines and the lung cancer risk is supported.

These findings replicate the observations of previous studies in Western populations and extend them to a general population in Korea. A study in the United States reported that both the National Cancer Institute—Maryland (NCI-MD) study and the Prostate, Lung, Colorectal, and Ovarian (PLCO) study reported increased odds ratios (ORs) for IL-6 (OR [95% CI]:3.29 [1.88–5.77] and 1.48 [1.04–2.10], respectively) and IL-8 (OR [95% CI]: 2.06 [1.19–3.57] and 1.57 [1.10–2.24], respectively) [11]. In a study conducted in Australia and Sweden, increased levels of IL-6 had an association with the lung cancer risk among former smokers (OR [95% CI]: 2.70 [1.55–4.70]) and current smokers (OR [95% CI]: 1.99 [1.15–3.44]). Similarly, elevated IL-8 levels were associated with an increased risk among former smokers (OR [95% CI]: 2.83 [1.18–6.75]) and current smokers (OR [95% CI]: 1.30 [0.69–2.44]). No significant associations were observed among never-smokers in this context [6]. Furthermore, a study by Pine et al., reported similar associations between elevated IL-6 and IL-8 levels and lung cancer risk across different racial groups, specifically among African Americans (IL-6 OR [95% CI]: 3.57 [1.94–6.58] and IL-8 OR [95% CI]: 2.20 [1.28–3.79]) and European Americans (IL-6 OR [95% CI]: 1.57 [1.07–2.30] and IL-8 OR [95% CI]: 1.55 [1.06–2.26]). Notably, elevated levels of IL-1β, IL-10, and TNF-α were linked to lung cancer risk only in the African American population [7]. Furthermore, soluble interleukin-6 receptor (sIL-6R) had a significant association with lung cancer risk among never-smoking Chinese women (OR [95% CI]: 2.37 [1.40–4.02]) [12].

The findings of this study, as reported in previous studies [7,11,13,14,15], indicate that serum IL-6 may be a potential universal biomarker for lung cancer, regardless of the histological type. However, IL-1β may be a specific biomarker for small-cell carcinoma, and IFN-γ may be a biomarker for squamous-cell carcinoma. This indicates that the mechanisms underlying lung cancer development may differ based on the histological type, highlighting the need for further in-depth research in this area. Furthermore, our study suggests that the mechanisms of action may significantly differ according to the cigarette smoking status. In other words, this implies that the etiology of lung cancer in never-smokers and ever-smokers may be distinct. While IL-6 seems to play a role in lung cancer development in both never-smokers and ever-smokers, the possibility that IL-1β and IFN-γ may play roles in the mechanism of lung cancer development in never-smokers cannot be ruled out.

The role of inflammation and immune mechanisms in tumorigenesis is intricate. The disruption of the balance between Th-1 and Th-2 cytokines leads to increased inflammation, which results in elevated oxygen and/or nitrogen free radicals, which is linked to cancer development [13]. Our study highlights the significant role of IL-6 in the universal mechanism of lung cancer development. Under conditions of inflammation, IL-6 is probably involved in facilitating tumorigenesis through a direct effect on lung epithelial cells via the nuclear factor κβ pathway [6,16,17]. Furthermore, IL-1β presents an alternative mechanism in lung cancer development. It has been revealed that IL-1β promotes carcinogenesis by repressing miR-101 expression through a cyclooxygenase 2 (COX2)/HIF1α pathway [18]. On the other hand, recent findings suggest that IFN-γ, previously known for its role in regulating tumor initiation and progression, plays a dual role by fostering the growth of tumor cells with immune-evasive properties within an immunosuppressive tumor microenvironment [19].

Despite the meaningful findings of this study, there are some limitations. First, we only obtained serum samples at the time of enrollment in the cohort, and serum levels of proinflammatory cytokines were assessed only once. Consequently, we could not account for changes in proinflammatory cytokines induced by lifestyle modifications, such as smoking habits (e.g., amount of smoking, tobacco types, favored tobacco products, quit smoking), alcohol consumption, dietary patterns, physical activity, and weight changes etc. Nevertheless, the associations among IL-6, IL-1β, and IFN-γ and the lung cancer risk in this study may not have been distorted, as these were not taken into account in both lung cancer cases and the subcohort. Second, our study encompasses several potential confounders, as occurs in most observational studies. To address this limitation, we conducted adjustments for age, sex, region, education achievement, cigarette smoking status, number of pack-years, and BMI. However, our findings might still be influenced by biases associated with other unmeasured confounders owing to the use of self-reported data and the potential existence of an unobserved confounder. Furthermore, in spite of our efforts to minimize the selection bias, the possibility for bias may persist. Third, the KNCCC cohort might not fully represent the Korean general population. However, it is important to emphasize that prospective cohort studies play a critical role in determining causal relationships between risk factors and diseases and may not necessarily require complete representativeness [20]. Finally, the current study had limited statistical power, resulting in risk estimates with wide confidence intervals, which meant that the exploration of interactions with well-known risk factors like cigarette smoke was limited. Therefore, larger-scale studies are needed to validate our results.

## 5. Conclusions

In conclusion, the findings of this study provide clinically valuable insights beyond the conventional questionnaires on tobacco history [20]. In line with previous studies, this investigation suggests the associations among serum levels of IL-6, IL-1β, and IFN-γ and the lung cancer risk, underscoring the potential of these cytokines to act as risk biomarkers. The utilization of these biomarkers for risk prediction holds the promise of facilitating the identification of the high-risk population, particularly for individuals who may benefit from low-dose computed tomography (LDCT) lung cancer screening. Moreover, these findings can serve as baseline evidence for recommendations to promote an anti-inflammatory lifestyle on prevention lung cancer in the general population.

## Figures and Tables

**Table 1:** Demographic characteristics of lung cancer cases and the subcohort.

		Lung Cancer Cases (n = 136)	Subcohort (n = 822)
Subcohort person-time (years)		939.88	9004.12
Major histologic types, n (%)	Squamous-cell carcinoma	34 (25.00)	
	Adenocarcinoma	39 (28.68)	
	Small-cell carcinoma	24 (17.65)	
	Others	39 (28.68)	
Age, years, median (IQR)		66.00 (61.00–70.00)	62.00 (52.00–68.00)
Sex, n (%)	Men	90 (66.18)	329 (40.02)
	Women	46 (33.82)	493 (59.98)
Region, n (%)	San-cheong	49 (36.03)	441 (53.65)
	Ui-ryeong	11 (8.09)	27 (3.28)
	Chang-won	14 (10.29)	121 (14.72)
	Choon-cheon	10 (7.35)	62 (7.54)
	Choong-joo	18 (13.24)	76 (9.25)
	Ham-an	34 (25.00)	95 (11.56)
Year of enrollment, n (%)	2001	23 (16.91)	72 (8.76)
	2002	6 (4.41)	16 (1.95)
	2003	28 (20.59)	107 (13.02)
	2004	33 (24.26)	147 (17.88)
	2005	11 (8.09)	103 (12.53)
	2006	19 (13.97)	129 (15.69)
	2008	11 (8.09)	104 (12.65)
	2009	2 (1.47)	73 (8.88)
	2010	3 (2.21)	71 (8.64)
Educational achievement	Elementary school or less	37 (27.21)	208 (25.30)
n (%)	Middle school	85 (62.50)	472 (57.42)
	High school	12 (8.82)	109 (13.26)
	College or more	2 (1.47)	33 (4.01)
Cigarette smoking status,	Never-smokers, n (%)	36 (26.47)	520 (63.26)
n (%)	Former smokers, n (%)	21 (15.44)	140 (17.03)
	Current smokers, n (%)	79 (58.09)	162 (19.71)
	Pack-years ^†^, median (IQR)	40.00 (20.00–49.00)	27.88 (15.00– 42.00)
Alcohol consumption status,	Non-drinkers	60 (44.12)	477 (58.03)
n (%)	<24 g per day	40 (29.41)	211 (25.67)
	≥24 g per day	36 (26.47)	134 (16.30)
BMI, kg/m^2^, median (IQR)		22.40 (20.56–24.22)	23.84 (21.41–25.97)

IQR, interquartile range; BMI, body mass index. ^†^ Calculated for former and current smokers.

**Table 2:** Serum levels of proinflammatory cytokines stratified by lung cancer cases and the subcohort.

			Lung Cancer Cases				Subcohort						
	n *	Min	25th	Median	75th	Max	n *	Min	25th	Median	75th	Max	%<LOD
IL-6, pg/mL	132	LOD	1.76	2.90	4.34	13.27	811	LOD	0.82	1.95	3.49	158.33	11.56
TNF-α, pg/mL	133	LOD	1.87	4.03	6.20	18.27	816	LOD	1.04	3.23	5.58	25.51	15.28
IL-1β, pg/mL	129	LOD	LOD	0.09	0.89	10.37	799	LOD	LOD	LOD	0.68	47.89	53.99
IFN-γ, pg/mL	128	LOD	LOD	0.05	1.83	11.24	792	LOD	LOD	LOD	1.39	63.06	60.43
IL-10, pg/mL	113	LOD	0.02	0.32	0.65	4.22	769	LOD	LOD	0.29	0.70	11.61	25.96

Abbreviations: LOD, limit of detection; IL-6, interleukin 6; TNF-α, tumor necrosis factor-α; IL-1β, interleukin; IFN-γ, interferon gamma; IL-10, interleukin 10. * Due to a shortage in the quantity, the final dual tests in some samples could not be conducted, causing them to be excluded from the statistical analysis. Thus, the sample size (n) varies somewhat for each substance.

**Table 3:** Associations between serum levels of proinflammatory cytokines and the lung cancer risk by quartiles.

				Model 1		Model 2		Model 3	
	Range (pg/mL)	Cases (n)	Subcohort Person-Time (Years)	HR (95% CI)	*p*-Value	HR (95% CI)	*p*-Value	HR (95% CI)	*p*-Value
IL-6									
1st quartile	<0.82	5	2391.28	Reference		Reference		Reference	
2nd quartile	0.82–1.95	33	2157.60	6.25 (2.44, 16.01)	<0.001	6.01 (2.34, 15.42)	<0.001	8.54 (3.30, 22.11)	<0.001
3rd quartile	1.95–3.49	41	2190.88	6.95 (2.74, 17.62)	<0.001	6.46 (2.54, 16.42)	<0.001	5.56 (2.17, 14.27)	<0.001
4th quartile	3.49–158.33	53	2135.01	8.17 (3.25, 20.50)	<0.001	8.24 (3.28, 20.75)	<0.001	9.51 (3.72, 24.30)	<0.001
TNF-α									
1st quartile	<1.04	24	2193.98	Reference		Reference		Reference	
2nd quartile	1.04–3.23	28	2249.59	1.14 (0.66, 1.97)	0.638	0.97 (0.56, 1.68)	0.920	0.86 (0.48, 1.52)	0.594
3rd quartile	3.23–5.58	40	2190.9	1.54 (0.93, 2.55)	0.098	1.40 (0.84, 2.33)	0.196	1.12 (0.65, 1.92)	0.693
4th quartile	5.58–25.51	41	2301.98	1.49 (0.90, 2.47)	0.121	1.25 (0.75, 2.08)	0.386	0.89 (0.51, 1.53)	0.665
IL-1β									
<LOD	<0.01	60	4911.91	Reference		Reference		Reference	
≥LOD	≥0.01	69	3832.00	1.44 (1.02, 2.04)	0.039	1.44 (1.02, 2.04)	0.038	1.61 (1.11, 2.32)	0.012
IFN-γ									
<LOD	<0.01	62	5606.81	Reference		Reference		Reference	
≥LOD	≥0.01	66	3060.48	1.76 (1.24, 2.49)	0.002	1.72 (1.21, 2.44)	0.002	1.85 (1.26, 2.72)	0.002
IL-10									
1st quartile	<0.01	26	2113.26	Reference		Reference		Reference	
2nd quartile	0.01–0.29	29	2049.87	1.15 (0.68, 1.96)	0.606	1.16 (0.68, 1.97)	0.591	1.07 (0.62, 1.86)	0.804
3rd quartile	0.29–0.70	31	2119.16	1.16 (0.69, 1.96)	0.575	1.18 (0.70, 1.99)	0.541	1.08 (0.62, 1.88)	0.782
4th quartile	0.70–11.61	27	2131.01	1.01 (0.59, 1.74)	0.964	0.95 (0.56, 1.64)	0.860	0.90 (0.51, 1.60)	0.722

Abbreviations: LOD, limit of detection; IL-6, interleukin 6; TNF-α, tumor necrosis factor-α; IL-1β, interleukin; IFN-γ, interferon gamma; IL-10, interleukin 10. Model 1 was unadjusted; Model 2 was adjusted for sex; Model 3 was further adjusted for year of enrollment, region, education achievement, cigarette smoking status (never, former, and current), number of pack-years (continuous variable), alcohol consumption status (non-drinkers, <24 g per day, and ≥24 g per day), and BMI (continuous variable).

**Table 4:** Lung cancer risk for each unit increase in natural log-transformed proinflammatory cytokines stratified with the cigarette smoking status.

	Never-Smokers (36 /5802.08) ^†^			Ever-Smokers (100/3202.04) ^†^		
	Median (IQR), (pg/mL)	HR (95% CI)	*p*-Value	Median (IQR), (pg/mL)	HR (95% CI)	*p*-Value
IL6	1.88 (0.78–3.46)	1.31 (1.04, 1.66)	0.022	2.32 (1.22–4.05)	1.38 (1.16, 1.64)	<0.001
TNF-α	3.06 (0.73–5.41)	1.09 (0.94, 1.27)	0.250	3.61 (1.93–6.04)	0.95 (0.85, 1.07)	0.423
IL-1β	0.01 (0.01–0.63)	1.25 (1.06, 1.48)	0.009	0.01 (0.01–0.74)	1.03 (0.94, 1.13)	0.533
IFN-γ	0.01 (0.01–1.07)	1.23 (1.07, 1.42)	0.004	0.01 (0.01–1.83)	1.08 (1.00, 1.16)	0.064
IL-10	0.25 (0.01–0.67)	1.03 (0.85, 1.24)	0.763	0.36 (0.02–0.72)	0.95 (0.85, 1.07)	0.391

^†^ n of cases/subcohort person-time (years). Adjusted for sex, year of enrollment, region, education achievement, cigarette smoking status (never, former, and current), number of pack-years (continuous variable), alcohol consumption status (non-drinkers, <24 g per day, and ≥24 g per day), and BMI (continuous variable). Abbreviations: IL-6, interleukin 6; TNF-α, tumor necrosis factor-α; IL-1β, interleukin; IFN-γ, interferon gamma; IL-10, interleukin 10.

## Data Availability

The data presented in this study are available in this article.
